# PKCα expression is a marker for breast cancer aggressiveness

**DOI:** 10.1186/1476-4598-9-76

**Published:** 2010-04-14

**Authors:** Gry Kalstad Lønne, Louise Cornmark, Iris Omanovic Zahirovic, Göran Landberg, Karin Jirström, Christer Larsson

**Affiliations:** 1 Center for Molecular Pathology, Department of Laboratory Medicine, Lund University, Malmö University Hospital, SE- 205 02 Malmö, Sweden

## Abstract

**Background:**

Protein kinase C (PKC) isoforms are potential targets for breast cancer therapy. This study was designed to evaluate which PKC isoforms might be optimal targets for different breast cancer subtypes.

**Results:**

In two cohorts of primary breast cancers, PKCα levels correlated to estrogen and progesterone receptor negativity, tumor grade, and proliferative activity, whereas PKCδ and PKCε did not correlate to clinicopathological parameters. Patients with PKCα-positive tumors showed poorer survival than patients with PKCα-negative tumors independently of other factors. Cell line studies demonstrated that PKCα levels are high in MDA-MB-231 and absent in T47D cells which proliferated slower than other cell lines. Furthermore, PKCα silencing reduced proliferation of MDA-MB-231 cells. PKCα inhibition or downregulation also reduced cell migration *in vitro*.

**Conclusions:**

PKCα is a marker for poor prognosis of breast cancer and correlates to and is important for cell functions associated with breast cancer progression.

## Background

Breast cancer is a heterogeneous disease which encompasses several subgroups with different morphology, genetic changes, and response to therapies [1,2]. It is therefore important to gain more insight into relevant therapeutic targets for each subgroup to optimize tailored treatment protocols for individual patients. Numerous intracellular signaling proteins have been suggested to be promising targets for blocking the malignancy of breast cancer cells. The protein kinase C (PKC) isoforms are examples of such potential therapeutic targets.

PKC is a family of serine/threonine kinases involved in several processes including proliferation, differentiation, apoptosis, and migration. The PKC isoforms are divided into three subgroups depending on the structure of the regulatory domain: classical (PKCα, βI, βII, and γ), novel (PKCδ, ε, and θ), and atypical (PKCζ and ι/λ) isoforms. Classical and novel PKCs contain a diacylglycerol (DAG)-binding C1 domain and are therefore regulated by activation of pathways that lead to DAG generation. Atypical PKCs are DAG-insensitive and regulated in a different manner [3].

Several studies have implicated the DAG-sensitive classical and novel PKC isoforms in promoting malignant features of breast cancer cells. PKCα has been coupled to estrogen receptor (ER) negativity [4] and estrogen-independent growth of cultured cells [5,6] and patients with PKCα-negative tumors had better response to endocrine treatment compared to patients with PKCα-positive tumors [7,8]. Moreover, increased PKCα expression leads to a more aggressive phenotype [4] and is associated with resistance to cytostatic drugs in MCF-7 cells [9,10]. PKCα is also evaluated as a therapeutic target for breast cancer [11]. However, PKCα levels are reduced in breast cancer compared to normal breast tissue [12,13]. Thus, there is evidence for both a promoting and a suppressing role for PKCα in breast cancer.

The role of PKCδ in breast cancer is ambiguous. Patients with PKCδ-positive tumors show better endocrine response compared to patients with PKCδ-negative tumors [8] and PKCδ has been shown to be crucial for UV light-induced apoptosis of cultured breast cancer cells [14]. However, several studies point to a pro-tumorigenic role of PKCδ in breast cancer. PKCδ can induce resistance to tamoxifen and irradiation in cultured breast cancer cells [15,16] and has been shown to promote both metastasis [17-19] and proliferation [20] of murine mammary cancer and epithelial cells. We have recently shown that depletion of PKCδ is sufficient to drive breast cancer cells into apoptosis [21].

PKCε has frequently been assigned oncogenic effects in breast cancer. Expression levels of PKCε have been shown to correlate with tumor grade, HER2 expression, ER negativity, and poor survival in breast cancer patients. Moreover, in MDA-MB-231 breast cancer cells, downregulation of PKCε reduced the tumor growth and metastatic capacity in mice [22]. There is also evidence that PKCε protects cells against apoptotic insults [23-25].

Taken together, the available *in vitro *and *in vivo *data highlight PKCα, PKCδ, and PKCε as future candidates for targets in breast cancer therapy and as markers for disease prognosis. However, so far there is limited knowledge on the potential of the different isoforms as diagnostic and prognostic markers in breast cancer. This study sheds light on this issue by analyzing the expression levels of these PKC isoforms in primary breast cancer tissue and our results indicate that PKCα is a potential marker of breast cancer aggressiveness.

## Methods

### Cell culture

All cell lines were obtained from ATCC. MCF-7, MDA-MB-231, and MDA-MB-468 breast cancer cells were maintained in RPMI 1640 medium (Sigma) supplemented with 10% fetal bovine serum (FBS; Invitrogen), 1 mM sodium pyruvate (PAA laboratories Gmbh), 100 IU/ml penicillin, and 100 μg/ml streptomycin (both Gibco). T47D cells were grown in DMEM supplemented with 10% FBS, 10 mM HEPES (PAA laboratories Gmbh), 100 IU/ml penicillin, and 100 μg/ml streptomycin. The media for MCF-7 and T47D cells were additionally supplemented 0.01 mg/ml insulin (Novo Nordisk A/S).

### Transfections

For siRNA transfections, cells were seeded at 35-50% confluency and grown in complete medium without antibiotics for 24 hours. Cells were transfected for 48 hours using 4 μl/ml Lipofectamine 2000 (Invitrogen) and 40 nM siRNA (Invitrogen, table 1) in Optimem (Gibco) according to supplier's protocol.

**Table 1:** siRNA oligonucleotides

siRNA	Oligonucleotides
Control	GACAGUUGAACGUCGAUUUGCAUUG
PKCα #1	CCGAGUGAAACUCACGGACUUCAAU
PKCα #2	CCAUCGGAUUGUUCUUUCUUCAUAA
PKCδ	CCAAGGUGUUGAUGUCUGUUCAGUA
PKCε	CACAAGUUCGGUAUCCACAACUACA

Plasmid transfections were carried out for five hours replacing normal medium with Optimem containing 2 μl/mL Lipofectamine 2000 and 2 μg/mL DNA according to supplier's protocol. Plasmids encoding PKC constructs fused to enhanced green fluorescent protein (EGFP) have been described previously [26].

### Tumor material

Cohort I originally consisted of tumors from 114 patients diagnosed with breast cancer at Umeå University Hospital and treated according to regional guidelines. The cohort is described in table 2. Due to lack of tumor material in the tissue microarray, 42-60 tumors could be analyzed, depending on the parameter investigated. Ki-67 had been classified in two groups <20% and >20% positive cells.

**Table 2:** Characteristics of the cohorts investigated

Cohort	I	II
Number of patients	114	512
Age at diagnosis, median (range)	60 (30-80)	65 (27-96)
Tumor size (mm), median (range)	22 (8-100)	16 (1-100)

Nodal status		
Positive	53	168
Negative	48	298
Missing	13	58

ER-status		
Positive	82	417
Negative	31	72
Missing	1	35

Cohort II included 512 consecutive breast cancer cases diagnosed at the department of Pathology, Malmö University Hospital, between 1988 and 1992. The cohort is described in table 2. Due to lack of tumor material in the tissue microarray, 223-263 tumors could be analyzed, depending on the parameter investigated. Ki-67 had been classified in three groups, 0-10%, 11-25% and 26-100% positive cells.

The cohorts represented a mix of all histological subtypes in proportions corresponding to the common incidence. The construction of tissue microarrays and clinicopathological properties of the cohorts have been described in detail elsewhere [27-32]. Ethical permissions were obtained from the Lund and Umeå Ethical Boards. The number of tumors that could not be evaluated for PKC expression due to lack of material is indicated as not evaluated in the tables.

### Cell pellet arrays

Cells were washed in phosphate-buffered saline (PBS) and fixed for 25 minutes in 4% paraformaldehyde in PBS with Mayer's hematoxylin (5 μl/ml) present during the last 5 minutes. The cells were pelleted, paraformaldehyde was removed and they were thereafter incubated over night in 70% ethanol followed by dehydration using increasing concentrations of ethanol and finally xylen. After dehydration, cell pellets were embedded in paraffin and arranged in a cell line array.

### Immunohistochemistry

Sections (4 μm) of the paraffin blocks were dried, deparaffinized, rehydrated and microwave-treated in 1× target retrieval solution with high pH (DAKO). All sections were stained in a DAKO Techmate™ machine and visualized using DAB. The antibodies used were PKCα (1:2000), PKCδ (1:1000), PKCε (1:400; all Santa Cruz Biotechnology, product numbers sc-208, sc-937 and sc-214), and Ki-67 (1:200; DAKO). All tissue microarray slides of a cohort were stained simultaneously with the same staining solutions ensuring identical conditions for each tumor. PKC stainings were scored according to cytoplasmic staining intensity were 0 representing lack of staining, 1 low staining, 2 moderate staining, and 3 strong staining. All cohorts were examined independently by two investigators and disconcordant results were re-evaluated. For Ki-67 analyses of breast cancer cell lines, Ki-67 staining intensity was scored as negative-low or moderate-strong staining.

### Sample preparation and Western blot

Cells were washed twice in ice-cold PBS and lyzed with RIPA buffer (10 mM Tris-HCl pH 7.2, 160 mM NaCl, 1% Triton-X 100, 1% sodium deoxycholate, 0.1% sodium dodecyl sulfate, 1 mM EDTA, 1 mM EGTA) supplemented with 40 μl/ml complete protease inhibitor (Roche Applied Science) for 30 minutes on ice. Lysates were cleared by centrifugation at 14,000 × *g *for 10 minutes at 4°C.

Proteins were separated with SDS-PAGE and transferred to polyvinylidene difluoride membranes (Millipore). Membranes were blocked with PBS containing 0.05% tween and 5% non-fat milk, and probed with antibodies towards PKCδ (1:500), PKCε (1:500), PKCα (1:3000), and actin (1:2000; MP Biomedicals, clone C4). Proteins were visualized with horseradish peroxidase-labeled secondary antibody (Amersham Biosciences) using the SuperSignal system (Pierce Chemical) as substrate. The chemiluminescence was detected with a CCD camera (Fuji Film).

### Analysis of cell growth (WST-1 assay)

Cell were seeded at a density of 2000 cells per well in 96-well culture plates, and incubated for 24 hours. For cell line comparison, viable cell number was measured 24 and 48 hours after seeding. For experiments with inhibition or activation of PKC, 2 μM Gö6976 (Calbiochem) or equal volume DMSO, or 16 nM 12-*O*-tetradecanoylphorbol-13-acetate (TPA; Sigma) was added in complete medium (CM) or serum-free medium (SFM) 24 hours after seeding, and cells were incubated for 72 hours prior to estimation of viable cell number. The amount of viable cells was assessed by a WST-1 cell viability assay (Roche Applied Science). Absorbance was measured in an ELISA plate reader Antos 2020 (Antos Labtech Instruments).

### Immunofluorescence and confocal microscopy

Immunofluorescence of PKCα was done as described [33] using Alexa Fluor 488-conjugated secondary antibodies. Cells were examined with a Zeiss LSM 710 confocal system using standard settings for Alexa Fluor 488.

### Cell cycle analysis

MDA-MB-231 cells were seeded at a density of 100,000 cells per 35 mm cell culture dish and transfected with siRNA. After transfection, cells were incubated in SFM or CM for 24 hours. Cells were trypsinized and fixed in 70% ethanol for 20 minutes at -20°C, washed in PBS, and incubated with a solution containing 3.5 μM Tris-HCl pH 7.6, 10 mM NaCl, 50 μg/ml propidium iodide (PI), 20 μg/ml RNase, and 0.1% igepal CA-630 for 20 minutes on ice in order to label DNA. 10,000 events were acquired on the FL-2 channel for the PI signal. Sample acquisition and analyses were performed with CellQuest software (Becton Dickinson).

### Wound healing assay

MDA-MB-231 or MCF-7 cells were seeded at a density of 150,000 cells per 35 mm cell culture dish and transfected with siRNA. After transfection, a scratch was made with a 200 μl pipette tip in a confluent area of the cell culture dish. Photographs of a selected area of each scratch were taken at indicated time points. For experiments with PKC inhibitors, MDA-MB-231 cells were seeded at a density of 350,000 cells per 35 mm cell culture dish and incubated for 24 hours before a scratch was made and PKC inhibitors were added. Photographs of a selected area of each scratch were taken 0 and 16 hours after scratching. The remaining wound area was measured using ImageJ software.

### Statistics

For TMA analysis, correlations between variables were calculated using Pearson's two-tailed significance test. Differences in distribution of various clinicopathological parameters in regard to PKC expression were also calculated using the χ^2^-test. The Kaplan-Meier analysis and the log rank test were used to illustrate differences between recurrence-free survival (RFS) and breast cancer-specific survival (BCSS) according to PKC expression. Cox regression proportional hazards models were used to estimate the impact of PKCα expression on breast cancer-specific survival in both univariate and multivariate analysis, adjusted for Nottingham histological grade (NHG), age, lymph node status, and tumor size of cohort II. For *in vitro *experiments, the significance of difference was assessed by analysis of variance (ANOVA) followed by Duncan's multiple range test. The difference was considered significant if the *p*-value was < 0.05. All statistical calculations were performed using SPSS V.11.0.

## Results

### PKC expression in breast cancer tumors

**Figure 1:**
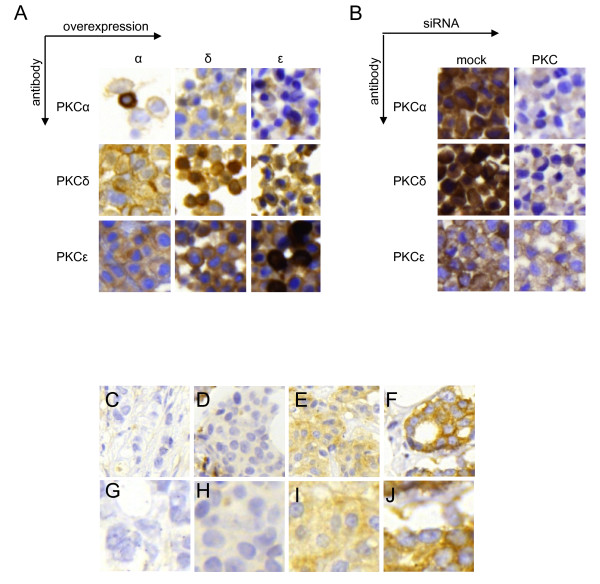
**Immunohistochemical stainings of primary breast cancer and validation of antibody specificity**. MCF-7 cells were transfected with vectors encoding PKCα (α), PKCδ (δ), or PKCε (ε) (A) and MDA-MB-231 cells were mock-treated or transfected with siRNA targeting PKCα (α), PKCδ (δ), or PKCε (ε) (B). Pellets of transfected cells were arranged in a cell line array and immunohistochemistry was performed with antibodies towards indicated PKC isoforms. (C-J) Examples of immunohistochemical staining of PKCα in breast cancer specimens from cohort II showing negative (C and G), low (D and H), moderate (E and I), and intense (F and J) staining with 20× magnification (C-F) and with 40× magnification (G-J).

Two cohorts of primary breast cancers (see experimental procedures for details) were analyzed for expression of PKC isoforms. Several batches of antibodies were tested to identify antibodies that did not cross-react with other isoforms. Cross reaction is a notorious problem for analyses of PKC isoforms. For PKCα, PKCδ, and PKCε we had access to antibodies that did not cross-react with other isoforms (Figure 1A and 1B). As can be seen in Figure 1A there is only strong immunoreactivity in some of the cells where the cognate isoform has been overexpressed. In Figure 1B it can be seen that downregulation with siRNA abolishes or markedly diminishes the staining with the antibody towards the cognate isoform.

When present in a tumor, all of the PKC isoforms investigated were generally cytoplasmic and expressed in all of the tumor cells. Therefore, only the cytoplasmic staining intensity was accounted for in the analyses. Figure 1C-J show examples of tumors with different staining intensities of PKCα, from negative to strong staining.

Initially a TMA of a smaller cohort (cohort I) was evaluated (Table 3 and 4). There was a significant correlation between PKCα staining intensity and lack of ER (p < 0.001) and progesterone receptor (PR; p = 0.002) as well as with tumor grade (p = 0.001) and proliferation rate (Ki-67; p < 0.001). PKCα levels did not correlate significantly with other clinicopathological parameters such as local or distal metastases (Table 3). For PKCδ and PKCε there were no significant correlations with any of the clinicopathological parameters analyzed (Table 3).

**Table 3:** Associations between PKCα, PKCδ, and PKCε intensity and clinicopathological variables in cohort I and II.

Cohort	I			II	
**Variable**	**PKCα**	**PKCδ**	**PKCε**	**PKCα**	**PKCε**

NHG					
ρ	0.401	-0.049	-0.029	0.247	-0.011
p	**0.001**	0.719	0.833	**<0.001**	0.863
n	60	57	55	249	263

Estrogen receptor					
ρ	-0.482	0.210	-0.016	-0.334	0.068
p	**<0.001**	0.120	0.907	**<0.001**	0.273
n	59	56	54	244	261

Progesterone receptor					
ρ	-0.402	-0.081	-0.216	-0.280	0.062
p	**0.002**	0.554	0.120	**<0.001**	0.342
n	58	55	53	223	240

Ki-67 positivity					
ρ	0.535	0.229	0.280	0.295	-0.056
p	**<0.001**	0.144	0.069	**<0.001**	0.363
n	46	42	43	244	261

Nodal status					
ρ	-0.027	0.095	-0.002	0.029	0.028
p	0.842	0.495	0.987	0.666	0.667
n	56	54	52	224	233

Distant metastasis					
ρ	0.159	0.162	-0.064	0.090	0.037
p	0.226	0.227	0.642	0.159	0.556
n	60	57	55	247	261

ρ: Pearson's correlation coefficient. p < 0.05 in bold. n: number of tumor samples.

**Table 4:** Associations between PKCα, PKCδ, and PKCε intensity and histological type in cohort I and II.

Cohort I		Ductal	Lobular	Medullary	Mucinous		
**PKCα intensity**	0	25	3	0	2		
p* = 0.295	1	20	0	1	0		
	2	4	0	0	0		
	3	4	0	1	0		
	n.e.	42	3	0	2		
			
**PKCδ intensity**	0	12	0	0	1		
p* = 0.850	1	33	1	2	1		
	2	7	0	0	0		
	3	0	0	0	0		
	n.e.	43	5	0	2		
			
**PKCε intensity**	0	0	0	0	0		
p* = 0.800	1	12	0	1	0		
	2	29	1	1	2		
	3	9	0	0	0		
	n.e.	45	5	0	2		
			
**Cohort II**		**Ductal**	**Lobular**	**Medullary**	**Mucinous**	**Tubular**	**Mixed**

**PKCα intensity**	0	124	17	1	10	14	13
p*<0.001	1	34	4	5	2	3	3
	2	10	0	4	0	0	1
	3	3	0	2	0	0	0
	n.e.	168	52	3	5	17	17
	
**PKCε intensity**	0	2	1	0	0	0	0
p* = 0.328	1	38	9	7	4	5	5
	2	90	19	4	7	9	7
	3	41	5	1	1	1	7
	n.e.	168	39	3	5	19	15

*χ^2^-test, p < 0.05 in bold, n.e. = not evaluated

A larger cohort (cohort II; Table 3 and 4) was then analyzed for expression levels of PKCα, since this isoform correlated to several clinicopatohological parameters in the first cohort, and PKCε, since our data somewhat contradict a published study [22]. The results from cohort II corroborated the findings in cohort I, where tumors with high PKCα levels had a higher proliferation rate (p < 0.001), were ER (p < 0.001) and PR-negative (p < 0.001), and of a higher histological grade (p < 0.001). Furthermore, a χ^2 ^analysis demonstrated that there is not an equal distribution of tumors with high and low PKCα-levels in the different histological subgroups in cohort II (Table 4). The skewed distribution is related to the tumors of medullary subtype. A tumor was defined as medullary if it fulfilled the following criteria: 1) lymphocytoplasmic reaction, 2) microscopic circumscription, 3) a syncytial growth pattern, and 4) poorly differentiated nuclear grade and high mitotic rate. For this group two out of twelve investigated tumors (17%) had high PKCα expression levels whereas the corresponding number for all evaluated breast cancers was five of 250 (2%). Only 8% of the medullary tumors were PKCα-negative, compared to 72% for all tumors. Thus, there is an overrepresentation of medullary carcinomas among tumors with high PKCα levels. For PKCε, the findings were similar to cohort I, with no correlation to any relevant clinicopathological parameters.

### PKCα expression is associated with poor prognosis

**Figure 2:**
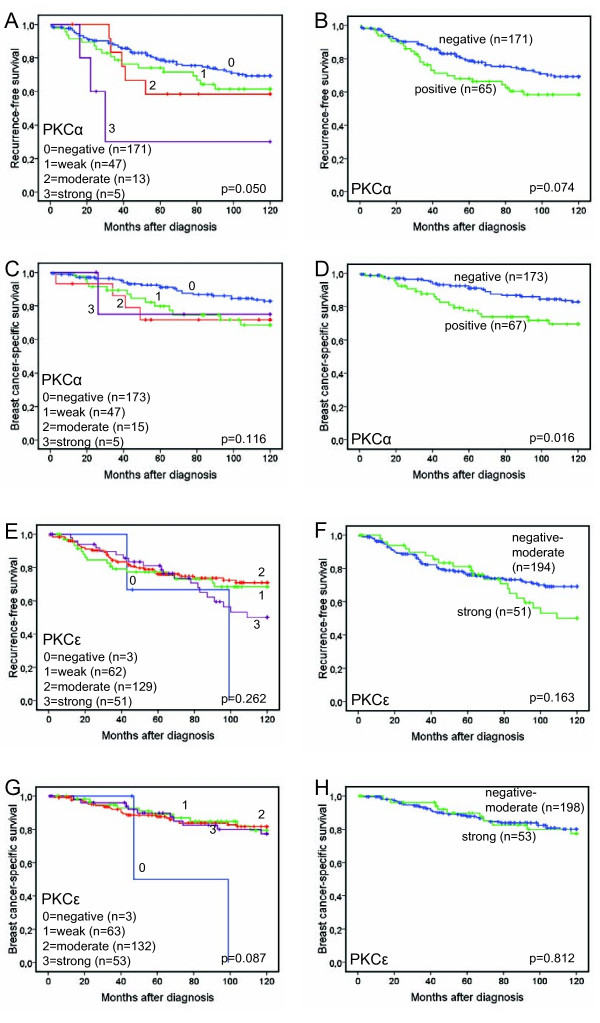
**Recurrence-free survival and breast cancer-specific survival according to PKCα and PKCε expression**. Kaplan-Meier estimates of 10 year recurrence-free survival (A-B and E-F) and breast cancer-specific survival (C-D and G-H) according to PKCα (A-D) and PKCε (E-H) expression.

We next analyzed the relationship between the expression of PKCα and PKCε and 10-year survival of the patients (Figure 2). As illustrated in figure 2A, lower PKCα levels were associated with a significantly prolonged 10-year RFS (p = 0.050). A similar trend was also seen for 10-year BCSS, but this did not reach statistical significance (Figure 2C; p = 0.116). Since the majority of the tumors are negative for PKCα and the other groups (staining intensity 1-3) are small, a dichotomized variable defined as absent staining versus any staining was used for the same analyses of recurrence-free and breast cancer-specific survival. When dichotomized, PKCα positivity was associated with a non-significant trend towards a poorer 10-year RFS, figure 2B; p = 0.074). However, patients with PKCα-negative tumors had a significantly improved 10-year BCSS compared to patients with PKCα-positive tumors (Figure 2D; p = 0.016). We also performed a Cox regression proportional hazards analysis, demonstrating estimates of relative risks (RR) according to PKCα expression in univariate and multivariate analyses, adjusted for age at diagnosis, tumor size, NHG, node status and ER expression (Table 5). This revealed that the association between PKCα positivity and a poor 10-year BCSS was independent of established prognostic parameters (multivariate RR = 2.123, 95% CI 1.092 to 4.126, p = 0.026).

**Table 5:** Cox univariate and multivariate analysis of breast cancer-specific survival in cohort II according to PKCα expression in all patients and when patients with medullary cancer are excluded.

	**All patients**		**Medullary cancers excluded**	
	
	**RR (95%CI)**	***p value***	**RR (95%CI)**	***p value***
	
	***Univariate***		***Univariate***	
PKCα negative	1.00		1.00	
PKCα positive	2.105 (1.148 to 3.862)	0.016	1.995 (1.069 to 3.721)	0.030
				
	***Multivariate***		***Multivariate***	
PKCα negative	1.00		1.00	
PKCα positive	2.123 (1.092 to 4.126)	0.026	1.978 (0.984 to 3.975)	0.055

Multivariate analysis adjusted for Nottingham histological grade (I-II/III), age (continuous), nodal status (0/1), tumor size (continuous), and ER (< or > 10%).

Since a majority of the medullary carcinomas analyzed were PKCα-positive and patients with these tumors generally have good prognosis [34] we also examined 10-year BCSS after excluding patients with medullary carcinomas and found that PKCα remained an independent prognostic factor (Table 5).

Recurrence-free or breast cancer-specific 10-year survival was not significantly influenced by PKCε expression, neither for all groups (staining intensity 0-3) nor when dichotomized into staining intensity 0-2 versus 3 (Figure 2E-H). A higher cutoff was chosen for dichotomization of PKCε due to a smaller proportion of negative tumors.

### PKC expression in breast cancer cell lines

**Figure 3:**
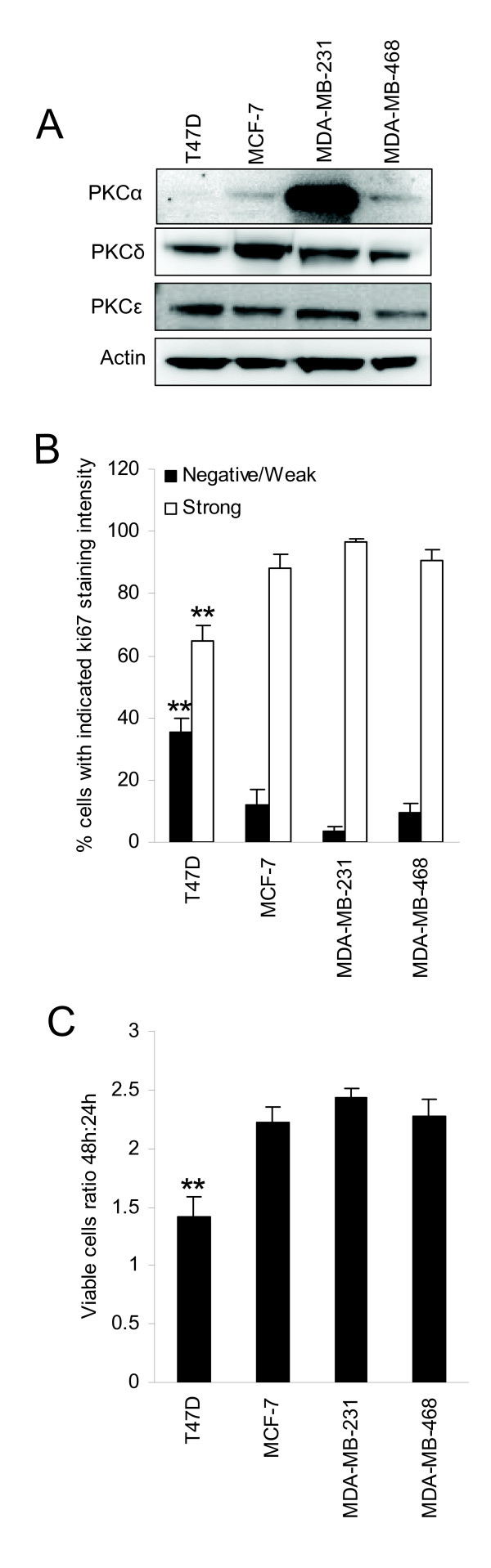
**PKC expression and proliferation of breast cancer cell lines**. Western blots show expression levels of PKCα, PKCδ, and PKCε in T47D, MCF-7, MDA-MB-231, and MDA-MB-468 breast cancer cells (A). Cell pellets of proliferating cells were immunohistochemically stained with anti-Ki-67. At least 200 cells per cell line were scored for negative-weak or strong staining intensity (B). The viable cell number was measured by WST-1 assay after 24 and 48 hours in culture. Ratio 48 h:24 h was used as an indicator of cell proliferation (C). For all experiments, cells were grown in complete medium. Western blots are representative of three independent experiments. Data in B and C are mean ± SEM, n = 3. Asterisks indicate statistically significant differences (* *p *< 0.05 and ** *p *< 0.01) compared to other cell lines (B and C).

To evaluate whether different breast cancer cell lines may represent the PKC isoform expression pattern in tumors, we measured PKC levels in four breast cancer cell lines (Figure 3A). High PKCα levels were seen in MDA-MB-231 cells compared to MCF-7 and MDA-MB-468 cells. PKCα was undetectable in T47D cells. PKCδ levels were higher in MCF-7 cells, whereas for PKCε no major differences in expression levels could be noted.

To investigate whether the relationship between PKCα and a high proliferation rate observed in the tumors can be seen in cell lines as well, we examined the proliferation rate of the cell lines (Figure 3B and 3C). T47D, which had no detectable PKCα, was the least proliferative cell line, supporting the tumor data. On the other hand, for MDA-MB-231 which clearly had the highest PKCα levels, 97% of the cells had moderate or strong Ki-67 staining intensity (Figure 3B). For the cell lines with lower but detectable PKCα levels, MCF-7 and MDA-MB-468, the corresponding numbers were 88% and 91%, respectively. This did not differ significantly from MDA-MB-231 cells which would not be expected considering that the fraction of positive cells was close to 100% in these cell lines. The Ki-67 data were supported by the growth rate of the cell lines as estimated by a ratio of viable cell number after 48 h in culture with the number obtained after 24 h (Figure 3C).

### PKCα is crucial for breast cancer cell proliferation under serum-free conditions

**Figure 4:**
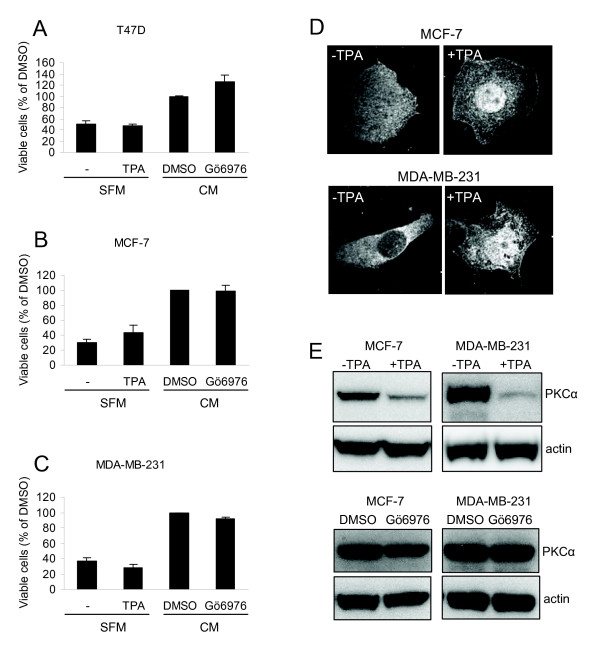
**Cell growth analysis after activation or inhibition of PKC**. T47D (A), MCF-7 (B), and MDA-MB-231 (C) breast cancer cells were incubated in the absence or presence of 16 nM TPA in serum free medium (SFM) or 2 μM Gö6976, GF109203X, or equal volume of DMSO in complete growth medium (CM) for 72 hours prior to WST-1 assay. Data (mean ± SEM, n = 3) represent the amount of viable cells expressed as percent of viable cells obtained under control conditions. The localization of PKCα in control or TPA-treated MCF-7 and MDA-MB-231 cells was examined with immunofluorescence followed by confocal microscopy (D). The levels of PKCα following treatment with TPA or Gö6976 were analyzed by Western blot (E).

To further examine whether PKCα is important for proliferation of breast cancer cells, the effects of a PKC activator and an inhibitor were studied (Figure 4A-C). Under serum-free conditions, activation of PKC by TPA was not sufficient to increase cell growth, as indicated by the amount of viable cells which represents the summative effect of proliferation and cell death. TPA did influence PKCα as seen by a relocation of PKCα to the plasma membrane and enrichment in the nucleus of both MCF-7 and MDA-MB-231 cells (Figure 4D). This indicates that PKCα is activated but the total levels of PKCα were also reduced following TPA treatment (Figure 4E) which complicates the estimation of the net effect of TPA on PKCα in the cells.

Gö6976, an inhibitor of classical PKCs, did not significantly reduce the number of cells indicating that PKCα activity is not critical for the growth of breast cancer cells under normal conditions.

**Figure 5:**
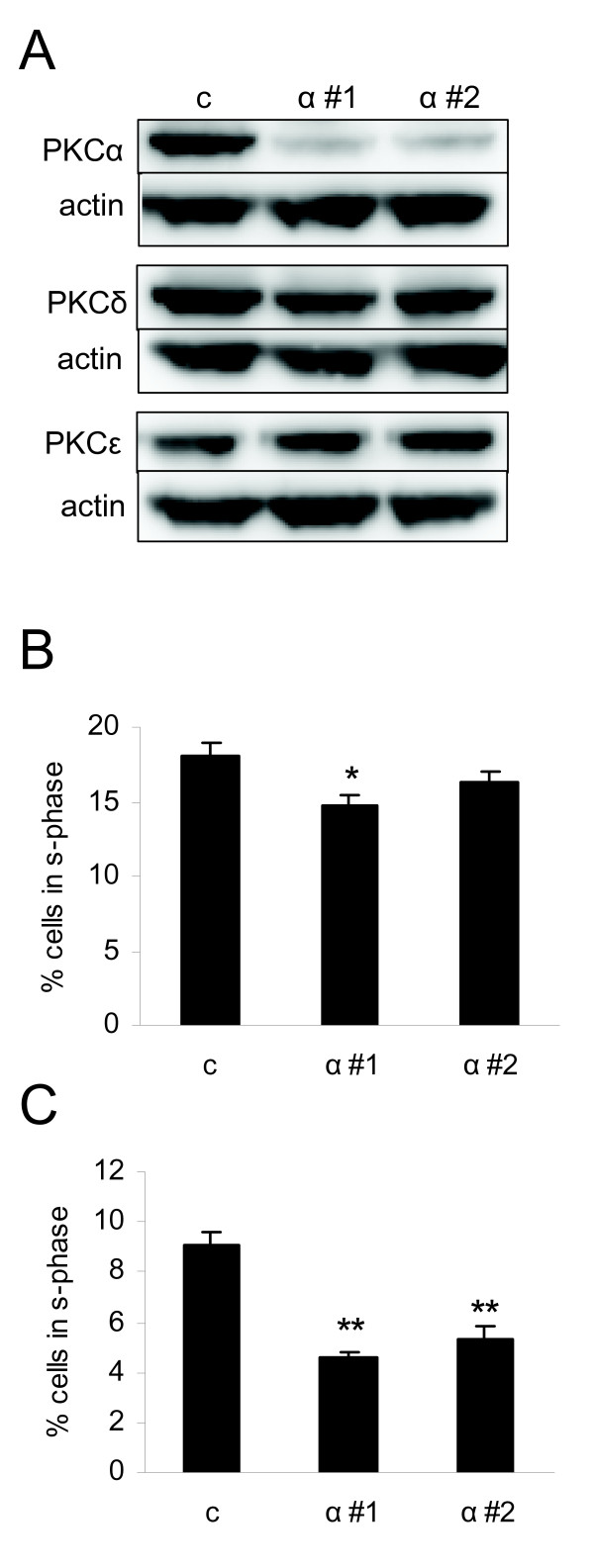
**Cell cycle distribution of MDA-MB-231 cells with downregulated PKCα**. MDA-MB-231 cells were transfected with two different siRNAs targeting PKCα (α #1 and α #2) or a control oligonucleotide. After transfection, cells were incubated with complete medium (CM) or serum-free medium (SFM) for 24 hours. Adherent cells were thereafter subjected to Western blot (A) or propidium iodide staining and flow cytometry (B and C). Western blots are representative of three independent experiments. Data in B and C (mean ± SEM, n = 3) show the percentage of cells in s-phase.

PKCα has been shown to promote proliferation independently of its catalytic activity [35]. This fact together with the fact that both TPA and Gö6976 can have unspecific effects beside modulation of PKCα activity led us to more specifically analyze the role of PKCα by using siRNA oligonucleotides followed by analysis of cell cycle distribution. PKCα levels were downregulated in MDA-MB-231 cells with both siRNAs targeting PKCα and this did not influence the expression levels of the other PKC isoforms investigated (Figure 5A). Only one of the PKCα oligonucleotides (α #1) significantly influenced the cell cycle distribution when cells were grown in serum-containing complete medium (CM; Figure 5B). The oligonucleotide slightly decreased the amount of cells in S-phase compare to control. However, under serum-free medium (SFM) downregulation of PKCα with either siRNA led to significantly decreased amount of cells in S-phase compared to control cells (Figure 5C). The result indicates that PKCα plays an important role for proliferation of cells particularly under sub-optimal conditions. Under optimal growth conditions PKCα may be redundant for proliferation.

### PKCα activity is necessary for migration of breast cancer cells

**Figure 6:**
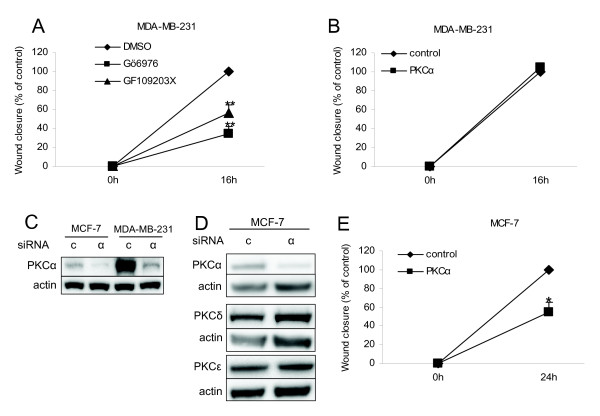
**Migration of MDA-MB-231 and MCF-7 cells**. MDA-MB-231 cells were subjected to wound-healing assay in presence or absence of PKC inhibitors (A) or after transfection with siRNA targeting PKCα (α) or a control oligonucleotide (c) (B). PKCα was downregulated in MCF-7 cells (C and D) before wound-healing assay was performed (E). Data (mean ± SEM, n = 4) show wound closure compared to closure in control conditions. Asterisks indicate statistically significant differences (* *p *< 0.05 and ** *p *< 0.01) compared to control conditions.

In breast cancer, PKCα has been associated with an increased ability of cells to migrate, since overexpression of PKCα has been shown to promote migration and metastasis of breast cancer cells [4,36,37]. Based on this we aimed at examining the importance of endogenous PKCα for breast cancer cell motility. Wound healing assays were performed with MDA-MB-231 cells in the absence or presence of PKC inhibitors (Figure 6A). Inhibition of classical PKCs (Gö6976) or classical and novel PKCs (GF109203X) suppressed the wound closure to 34% and 56%, respectively, of the DMSO treated cells, indicating that PKC activity is important for the ability of MDA-MB-231 cells to migrate. Since PKC inhibitors are isoform-unspecific, we downregulated PKCα with siRNA in MDA-MB-231 cells prior to a wound-healing assay, to more specifically study the role of PKCα in migration. However, downregulation of PKCα did not affect the migration of MDA-MB-231 cells (Figure 6B). MDA-MB-231 cells have high basal levels of PKCα compared to the other cell lines investigated in this study (Figure 3A). Transfection of these cells with siRNA targeting PKCα might not sufficiently reduce the PKCα levels. A comparison with MCF-7 cells (Figure 6C) actually demonstrates that following siRNA treatment of MDA-MB-231 cells the PKCα levels in these cells are roughly the same as in MCF-7 cells. Therefore, to obtain a more substantial PKCα depletion we downregulated PKCα in MCF-7 cells and thereafter performed a wound healing assay (Figure 6E). PKCα silencing in MCF-7 cells did not influence the other PKC isoforms investigated (Figure 6D). In these cells downregulation of PKCα significantly reduced migration into the wound to 55% of the control cells (Figure 6E). The results suggest that PKCα activity is crucial for migration of breast cancer cells *in vitro*.

## Discussion

Several studies have suggested that DAG-sensitive PKC isoforms contribute to the progression of breast cancer and the malignant characteristics of breast cancer cells. In particular the PKCα, PKCδ, and PKCε isoforms have been highlighted as potential targets for therapy of breast cancer or of specific subsets of the disease. This led us to design this study in which the expression of these PKC isoforms in primary breast cancer tumors has been examined to assess their utility as markers of tumor aggressiveness.

To substantiate the analysis, two different cohorts of primary breast cancer tumors were used, and the results from the cohorts were similar. We found significant correlations between PKCα levels and several markers of tumor aggressiveness including ER negativity. A correlation between PKCα levels and ER negativity has also been observed in a recent study [8] using 70 tumors from patients that had received systemic endocrine therapy. They also showed that high PKCα levels predicted a worse outcome in response to endocrine therapy, which is also supported in an earlier study with a smaller number of patients [7]. Our data, as demonstrated in two separate cohorts, firmly establish the relationship between PKCα and ER negativity. In addition, we identify a clear correlation between PKCα and PR negativity, and a positive correlation with tumor grade and high proliferation rate, further supporting the notion that PKCα expression is associated with parameters related to tumor aggressiveness. Finally, PKCα expression predicts a worse disease outcome with a significantly poorer 10-year breast cancer-specific survival for patients with primary tumors that were PKCα-positive. The results from the multivariate analysis further indicate that PKCα is an independent prognostic factor in breast cancer.

However, other studies have indicated that PKCα levels actually are decreased in breast cancer compared to normal breast tissue [12,13]. This may not necessarily contradict our findings since a vast majority of the cancer samples in our material were essentially PKCα-negative, which is in line with the notion that PKCα downregulation is a common event during breast cancer progression. Therefore, the published data, together with our results, suggest that most breast cancers are PKCα-negative, but that there are smaller subgroups with higher PKCα levels, displaying more aggressive clinicopathological features. If PKCα is to be used as a target for breast cancer therapy these data highlight the need to evaluate PKCα levels prior to such intervention.

In the largest cohort (II) there was also a significant association of PKCα levels with histological subtypes. Cancers with medullary histology were over-represented in the group with high PKCα levels. In addition, the only medullary cancer in cohort I was PKCα-positive.

In line with the tumor data demonstrating that PKCα levels correlate with features of aggressiveness, an association of PKCα with malignant features has also been seen in breast cancer cell lines. Increased levels of PKCα correlate to and can induce tamoxifen [38,39] and multidrug [9,10] resistance of ER-positive cell lines. In addition, overexpression of PKCα in MCF-7 cells leads to increased proliferation which is in line with our tumor data [4], but also make them more susceptible to apoptotic insults [40,41]. We found that the cell line with no detectable PKCα expression, T47D, had a lower percentage of Ki-67-positive cells and slower growth rate than the other cell lines, similar to the tumor data. However, the cell line with the highest PKCα expression, MDA-MB-231, only had marginally higher Ki-67 positivity compared with cell lines with detectable but much lower expression. This may be related to the fact that the fraction of Ki-67-positive cells was high (97%) and could not be further elevated.

Neither inhibition of PKCα in the presence of serum nor PKC activation under serum-starvation influenced the growth of the cell lines in a manner that would support an essential role for PKCα for breast cancer cell growth. A recent paper has shown that the PKCα protein, but not activity, is essential for glioma cell proliferation [35]. We found that downregulation of PKCα with siRNA caused a modest effect on cell cycle distribution of MDA-MB-231 cells grown in complete medium. However, under serum-free conditions, PKCα silencing clearly reduced the amount of proliferating cells suggesting that, as in glioma cells, the PKCα protein, but maybe not its catalytic activity, supports proliferation under sub-optimal conditions.

Studies *in vitro *have demonstrated that increasing the PKCα expression in MCF-7 cells makes them more migratory in response to PKC activation [42]. Our experiments with cell lines indicate that PKC activity supports migration of MDA-MB-231 cells. However, downregulation of PKCα with siRNA in this cell line did not affect cell migration. MDA-MB-231 cells have high basal expression levels of PKCα and incomplete downregulation of PKCα may explain the lack of effect on cell migration. This assumption is supported by the more efficiently suppressed wound healing upon downregulation of PKCα in MCF-7 cells. Our data therefore indicate that PKCα activity is important for migration of breast cancer cells, in line with previous findings using PKCα overexpression. A migratory propensity might facilitate the metastasation of a tumor. However, our tumor data did not support a role for PKCα in dissemination. There was no correlation between PKCα expression and nodal or distant metastases. Thus, the effects of PKCα on migration may primarily be of importance *in vitro*.

For PKCδ there is less information regarding expression levels in primary tumors. One study has shown that low levels of PKCδ, particularly in combination with high PKCα, predict resistance to endocrine therapy [8]. In this study, we could not observe any significant associations between PKCδ expression and relevant clinicopathological parameters. Thus, altered PKCδ expression does not seem to be a prerequisite for breast cancer progression.

PKCε has been proposed to be a marker of aggressive breast cancer since its expression was reported to be elevated in hormone receptor-negative breast cancers with high tumor grade and *HER2 *amplification [22]. However, a relationship between PKCε expression and tumor grade could not be confirmed in this study, suggesting that it might not be apparent in cohorts representing all histological subtypes of breast cancer. It is possible that the level of PKCε is a marker of aggressiveness in more defined subgroups of breast cancer.

## Conclusions

In conclusion, our findings demonstrate that expression of PKCα correlates to both ER and PR negativity as well as high histological grade and proliferation rate in breast cancer. An important role for PKCα in breast cancer cell proliferation was also observed *in vitro*. PKCα expression is not associated with metastasis in breast cancer samples, but PKCα activity supports migration of breast cancer cells *in vitro*. Importantly, PKCα expression predicts for a poorer 10-year breast cancer-specific survival, independently of established prognostic parameters. Thus, we propose that PKCα might be a useful marker for prognostication and treatment stratification in breast cancer.

## Competing interests

The authors declare that they have no competing interests.

## Authors' contributions

GKL evaluated clinical samples, performed statistical analyses, participated in the design of and did most of the experimental work, and assembled the drafts of the manuscript.

LC did some experimental work.

IOZ took part in the evaluation of the antibodies and clinical samples.

GL supervised the analysis of cohort I and participated in interpretative discussions.

KJ supervised the analysis of cohort II and statistical analyses of patient data, and helped draft the manuscript.

CL conceived of the study, participated in the design of the experimental work and helped draft the manuscript.

All authors read and approved the final manuscript.
